# Cognitive functioning is prognostic in patients with IDH1-wild type and MGMT-unmethylated high-grade gliomas

**DOI:** 10.3325/cmj.2023.64.383

**Published:** 2023-12

**Authors:** Uroš Smrdel, Andreja Cirila Škufca Smrdel, Anja Podlesek, Marija Skoblar Vidmar, Gregor Kos, Jana Markovic, Jana Jereb, Jana Knific, Tina Rus

**Affiliations:** 1 Division of Radiotherapy, Institute of Oncology Ljubljana, Ljubljana, Slovenia; 2 Department of Psycho-Oncology, Institute of Oncology Ljubljana, Ljubljana, Slovenia; 3 Department of Psychology, Faculty of Arts, University of Ljubljana, Ljubljana, Slovenia

## Abstract

**Aim:**

To investigate the prognostic factors of survival in patients with high-grade gliomas without isocitrate dehydrogenase-1 (IDH) mutation and O 6 -methylguanine-DNA methyltransferase (MGMT) methylation.

**Methods:**

The study enrolled Slovenian patients with high-grade gliomas. Postoperatively, they completed a battery of neuropsychological tests. Demographics and clinical data were collected. The results of cognitive tests were converted to standardized scores and dichotomized based on impairment. A univariate Cox proportional hazard regression model was used to determine clinical predictors, and a multivariate Cox model was used to determine the prognostic value of cognitive test results. Kaplan-Meier curves were constructed, and survival was compared with the log rank test.

**Results:**

The study enrolled 49 patients with IDH wild-type, MGMT-unmethylated high-grade gliomas. The median time to progression was 9.92 months (7.25, 12.59) and the overall median survival was 12.19 months (8.95, 15.4). Age and the extent of surgery were significant prognostic factors for survival. After controlling for these factors, cognitive functioning in the domain of verbal fluency remained a significant predictor of survival outcomes.

**Conclusion:**

Cognitive functioning in the domain of verbal fluency was associated with overall survival independently of age and the extent of surgery. Cognitive functioning could be an important stratifying tool in this group of patients lacking other predictors.

Malignant gliomas comprise WHO-grade III astrocytomas and oligodendrogliomas, as well as WHO-grade IV gliomas (grade IV astrocytomas and glioblastomas). The grading has changed with the discovery of genetic and epigenetic markers with prognostic and predictive value, the most important of which are the isocitrate dehydrogenase-1 (IDH) mutation and O 6 -methylguanine-DNA methyltransferase (MGMT) methylation (1,2). The IDH1 mutation is present in about 5% (3%-12%) of primary glioblastomas and is more common in grade III tumors (up to 80%) (3). Patients with IDH1-mutated high-grade tumors (IDH1-mut) have a longer overall survival and a longer progression-free survival than patients without this mutation (IDH1-wt) (4). About 50% of glioblastoma patients have a methylated MGMT promoter region (MGMT-met). In these patients, radio-chemotherapy followed by chemotherapy improves overall and progression-free survival (5-7). In the majority of IDH-mutated gliomas, the MGMT promoter is also methylated, but *vice versa* is not true (8,9). MGMT is also methylated to varying degrees in other glial tumors, which translates into survival benefit (10).

Cognitive decline precedes radiological progression, and was found to be an independent prognostic factor for survival in high-grade (11,12) and low-grade brain tumor patients (13). However, most of these studies did not differentiate the patients based on IDH1 mutation and MGMT methylation status. Cognitive functioning has been shown to be better in patients with IDH1-mut tumors compared with IDH1-wt tumors (14-17). However, the group of patients with IDH-wt and MGMT-unmethylated (MGMT-unmet) tumors has been understudied. Therefore, it is important to determine factors related to treatment outcome in these patients and to identify a group that would benefit from a change in treatment strategy. The aim of this study is to investigate whether cognitive functioning has a prognostic value for survival in patients with IDH1-wt, MGMT-unmet high-grade gliomas.

## Patients and methods

### Patients and data collection

This prospective observational study enrolled patients with high-grade gliomas treated at the Institute of Oncology Ljubljana between March 2019 and December 2021. The diagnoses were confirmed histologically. Patients were referred to an oncologist after surgery at one of the two neurosurgical centers. At the time of referral, the patients consented to participate in the study. The study was approved by the Institutional Review Board of the Institute of Oncology Ljubljana and by The National Medical Ethics Committee of the Republic of Slovenia (0120- 393/2018/10, date 12/12/2018) and complied with the Declaration of Helsinki.

Exclusion criteria were a histological finding other than WHO grade III or grade IV glioma, Karnofsky performance status lower than 70%, inability to undergo evaluation, and age less than 18 years. Disease and treatment data were obtained from medical records. All patients also underwent a molecular and genetic analysis of the tumor tissue. Cognitive functioning was assessed by a clinical psychologist before systemic treatment.

### Cognitive functioning

Cognitive functioning was assessed with a battery of tests. In the verbal memory domain, we used the Verbal Learning Test (TBU), measuring immediate recall, delayed recall, and recognition (18). In the cognitive domain of verbal fluency, we used the Controlled Oral Word Association Test (SCOWA) (19). Visual-motor speed was assessed with the Trail Making Test Part A (TMT A) and executive functions were evaluated with the TMT B (20).

### Statistical analysis

Descriptive statistics were used to summarize demographic data. Cognitive test scores were converted into standardized scores (*z*-scores). For each patient, the percentage of impaired results on cognitive tests was assessed (cut point *z*≤-1.5 below the mean of the normative group). We then compared patients with an impaired result (*z*≤-1.5 or those who could not complete the test) with patients with a non-impaired result (*z*>-1.5).

In the initial base-univariate Cox proportional hazards model, we first included the known clinical predictors of survival. The predictors that proved to be statistically significant were included as variables in the Cox model to assess the prognostic value of cognitive test scores. Kaplan-Meier curves were constructed for the significant clinical predictors and for cognitive tests. Survival was compared with a log-rank test. The analysis was performed with SPSS, version 29 (IBM Corp., Armonk, NY, USA). All hypotheses were tested at a 5% alpha error rate (95% confidence intervals).

## Results

**Figure 1:**
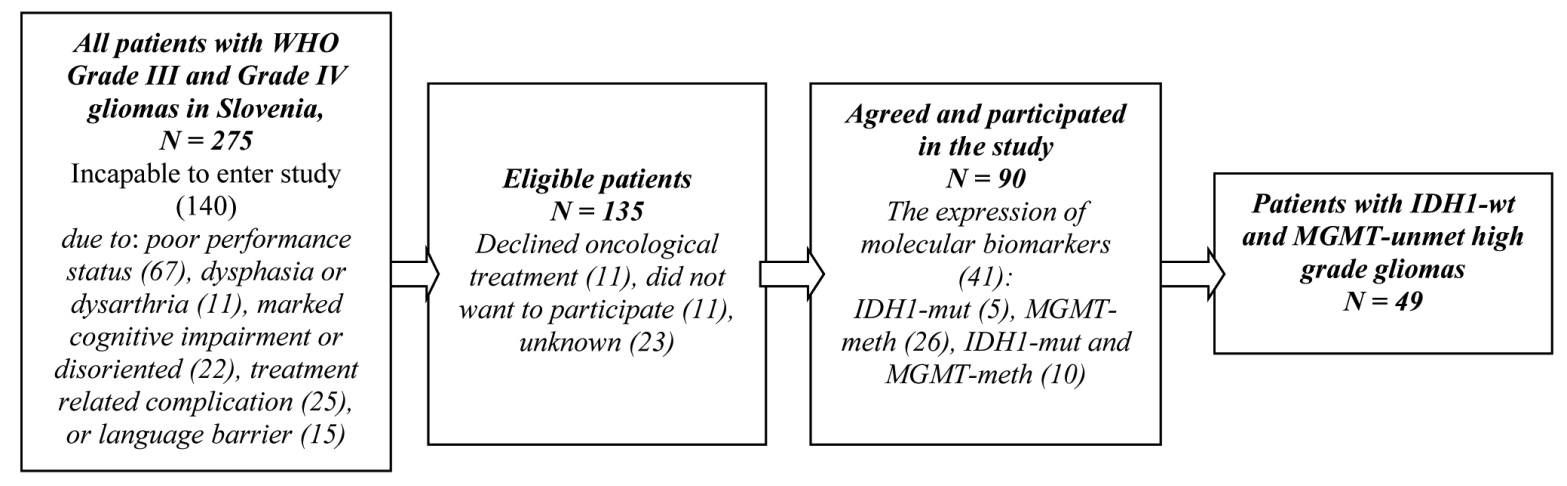
Recruitment protocol.

At the time of the study, 275 patients were diagnosed with a glial tumor, and 90 of them (33%) participated in the study (Figure 1). We analyzed the data from 49 patients with IDH1-wt and MGMT-unmet high-grade tumors. Among them, 48 patients had glioblastoma and one had anaplastic astrocytoma. Demographic, disease, and treatment data are shown in Table 1.

**Table 1:** Demographic, disease and treatment characteristics of patients with high-grade glioma (N = 49)

Demographic characteristic	
Age; mean (min/max/SD)	60.61 (31/84/9.34)
Age group; n (%)	
≤50 years	6 (12)
51-65 years	29 (59)
>65 years	14 (29)
Sex; n (%)	
female	16 (33)
male	33 (67)
Education level (years); n (%)	
≤9	8 (16)
10-13	27 (55)
≥14	14 (28)
Disease characteristics	
WHO grade n (%)	
III	1 (1)
IV	48 (98)
Karnofsky performance status; n (%)	
70	20 (41)
80	22 (45)
90	5 (0)
100	2 (4)
Tumor location; n (%)	
frontal	17 (35)
temporal	13 (26)
parietal	12 (25)
occipital	3 (6)
diffuse	2 (4)
central	2 (4)
Hemisphere	
right	19 (39)
left	25 (51)
left-right	5 (10)
Treatment characteristics	
Type of resection; n (%)	
biopsy	8 (16)
reduction	29 (59)
gross tumor resection	12 (24)
Weeks from surgery to testing; mean (min/max/SD)	5.80 (3/12/2.08)
Intention to treat; n	
yes	49
no	0
Corticosteroids; mg (min/max/SD)	5.63 (0/24/5.20)
yes	34
no	15
Epilepsy	
yes	12
no	37

The median time to progression was 9.92 months (7.25, 12.59), and the median overall survival was 12.19 months (8.95, 15.4). By the time of analysis, 35 patients had died. All patients were scheduled for chemo-radiotherapy, but not all of them started it. Radiotherapy was not started in 4 patients, and treatment was terminated early in 7 patients due to deterioration, progression, or death.

A large proportion of patients were unable to complete at least one neuropsychological test. The proportion was highest for the TMT B test, which measures executive functions (59%), and lowest for the verbal learning test SVLT and the SCOWA test of verbal fluency (12% for both). The proportion of patients who achieved an impaired result on cognitive tests (*z*≤-1.5 or unable to complete the test) was lowest in the verbal fluency domain (47%) and highest in the domain of executive functions (78%) (Table 2).

**Table 2:** Standardized test scores (z-values) and proportion of patients with impaired results on psychological cognitive functioning tests

Domain	Test abbreviation	*f* (%) of patients able to complete the test	Mean *z-*score^†^	SD of z-scores^†^	*f* (%) of patients with *z*≤-1.5	*f* (%) of patients with *z*≤-1.5 or unable to complete the test
Visual-motor speed	TMT A	34 (69)	2.25	2.37	19 (55)	34 (69)
Executive function	TMT B	20 (41)	1.66	2.09	9 (45)	38 (78)
Verbal fluency	SCOWA	43 (88)	-1.24	0.85	17 (39)	23 (47)
Memory		43 (88)				
Immediate recall	SVLT-ir		-1.89	0.98	27 (63)	33 (67)
Delayed recall	SVLT-dr		-1.99	1.24	26 (60)	32 (65)
Recognition	SVLT-recog		-2.56	2.49	22 (53)	28 (57)

*Abbreviations: TMT – Trail Making Test; SCOWA – Controlled Oral Word Association Test; SVLT-ir – memory test of immediate recall; SVLT-dr – memory test of delayed recall; SVLT-recog – memory test of recognition.

^†^Of those who completed the test.

**Figure 2:**
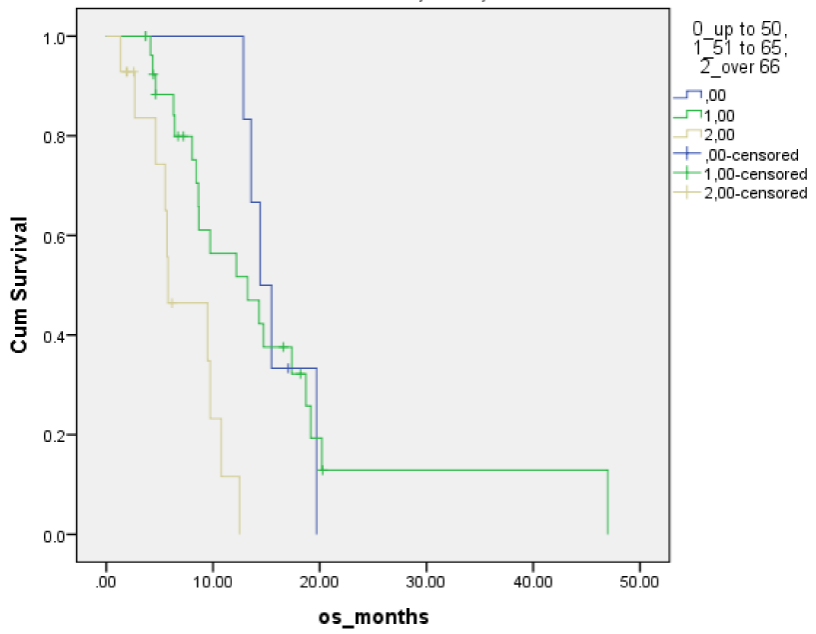
Kaplan-Meier curves of overall survival stratified by age categories.

In the initial Cox model, we included individual demographic and clinical variables as prognostic factors for overall survival (Table 3). Two variables were significantly associated with survival: age and the type of surgery. Older age was associated with shorter survival (*P* = 0.002). The median survival was 5.78 months (0.39, 11.17) in patients older than 65, 13.24 months (6.43, 20.05) in patients aged 50 to 65, and 14.42 (12.14, 16.71) in patients younger than 50 years. The Kaplan-Meier estimator showed significant differences in survival among the three age groups (log-rank test: χ^2^ (2) = 14.31, *P* = 0.001) (Figure 2). Compared with patients younger than 50 years, patients older than 65 years had a significantly higher risk of death (*P* = 0.005), while no differences were observed for patients aged 50 to 65 (*P* = 0.706).

**Table 3:** Cox proportional hazards models – demographic and clinical variables*

	b	SE_b_	df	p	HR (95% CI)
Sex	0.33	0.37	1	0.372	1.39(0.762, 2.89)
Age	0.06	0.02	1	0.002	1.06 (1.02, 1.11)
Age (categories)			2	0.003	
<50 years	reference				1
51-65 years	0.19	0.51	1	0.706	1.21 (0.44, 3.31)
>65 years	1.69	0.60	1	0.005	5.42 (1.66, 17.62)
Education level			2	0.568	
≤9 years	reference				
10-13 years	0.46	0.55	1	0.288	0.57 (0.19, 1.62)
≥14 years	-0.19	0.385	1	0.616	0.824(0.39, 1.75)
Performance status	0.02	0.02	1	0.315	0.98 (0.93, 1.02)
Use of corticosteroids	0.05	0.03	1	0.158	1.05 (0.98, 1.11)
Epilepsy status	0.22	0.43	1	0.600	0.78 (0.34, 1.85)
Time from surgery	0.00	0.01	1	0.866	0.99 (0.98, 1.02)
Type of surgery			2	0.014	
biopsy	reference				
reduction	1.08	0.46	1	0.020	0.34 (0.14, 0.61)
gross tumor resection	1.63	0.58	1	0.005	0.20 (0.06, 0.61)
Localization of tumor			3	0.411	
frontal	reference				
temporal	0.49	0.45	1	0.280	1.63 (0.67, 3.93)
parietal	-0.02	0.51	1	0.975	0.98 (0.36, 2.66)
other	0.79	0.58	1	0.175	2.203 (0.70, 6.90)
Hemisphere			2	0.084	
right	reference				
left	-.74	0.57	1	0.197	0.48 (0.15, 1.47)
left-right	-1.27	0.59	1	0.032	0.28 (0.90, 0.89)

*Abbreviations: SE – standard error; df – degrees of freedom; HR – hazard ratio.

†Each row presents results of a separate model that included the specific demographic or clinical variable.

**Figure 3:**
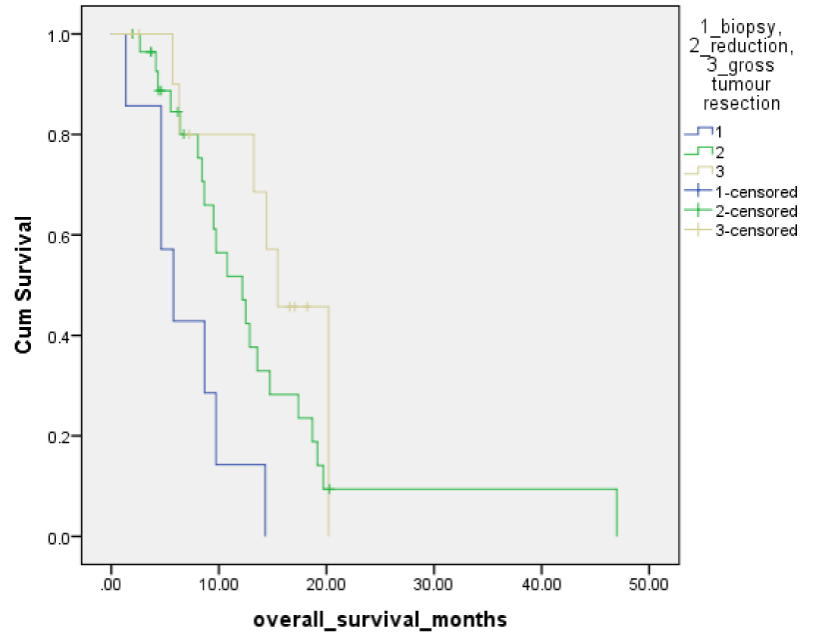
Kaplan-Meier curves of overall survival stratified by the type of surgery.

The type of surgery was also significantly associated with survival (*P* = 0.014). The median survival was 5.78 months (2.83, 8.73) in the biopsy group, 12.19 (8.11, 16.27) months in the reduction group, and 15.47 (12.18, 18.77) months in gross tumor resection group. Compared with the biopsy group, both the reduction (*P* = 0.020) and gross tumor resection (*P* = 0.005) groups had a reduced risk of death. The Kaplan-Meier curves (Figure 3) were significantly different (log rank test: χ^2^ (2) = 9.65, *P* = 0.008).

There was no relationship between survival and education level, sex, epilepsy status, performance status, localization of tumor, hemisphere, use of corticosteroids, and time from surgery (Table 3), so these factors were not further included in the model.

**Figure 4:**
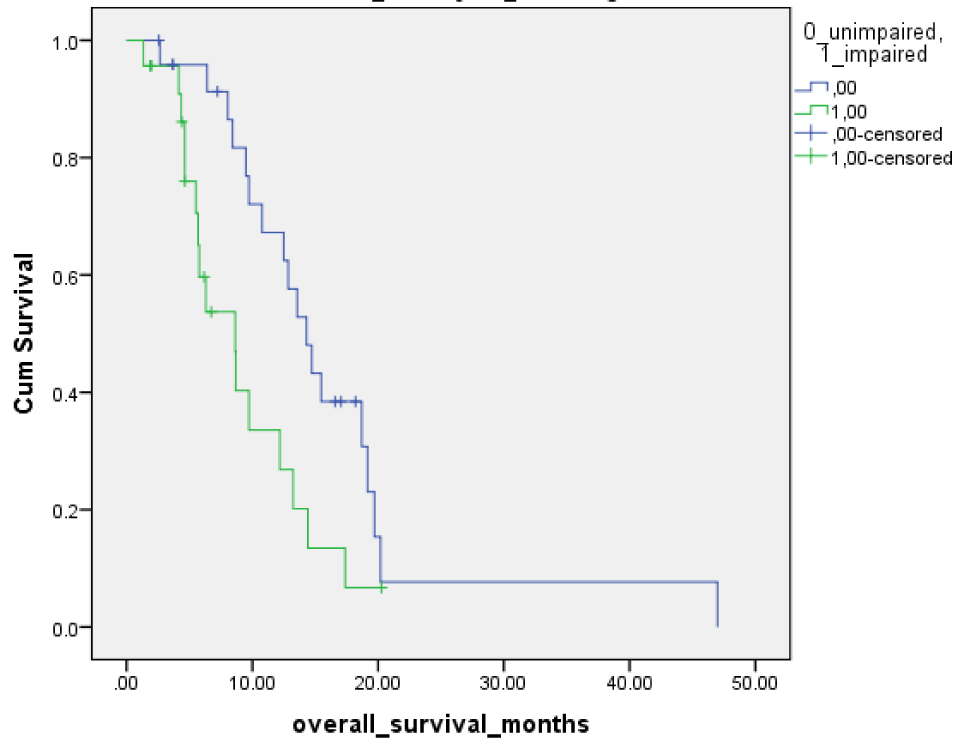
Kaplan-Meier curves of overall survival stratified by the results of the Controlled Oral Word Association Test (cognitive domain of verbal fluency).

In the analysis of the association between cognitive tests and survival, age and the type of surgery were included in the Cox model as covariates. The verbal fluency score was a significant predictor of survival (*P* = 0.028). No other test was associated with a greater risk of death; neither was the number of impaired tests (Table 4). Kaplan-Meier curves of survival stratified by impairment status on the SCOWA test are shown in Figure 4. The median survival was 14.32 (11.55, 17.10) months in the group with non-impaired test results and 8.64 (5.17, 12.11) months in the group with impaired test results. This difference in survival was significant (log-rank test: χ^2^ (1) = 5.43, *P* = 0.020).

**Table 4:** Cox proportional hazards models – cognitive tests^*†^

	Capable patients only Scores dichotomized based on impairment (*z*≤-1.5 vs *z*>-1.5)				All patients Scores dichotomized based on impairment (z≤-1.5 or inability to complete the test vs z>-1.5)			
Cognitive test	b	SE_b_	p	HR (95% CI)	b	SE	p	HR (95% CI)
TMT A	0.31	0.38	0.413	1.37 (0.64,2.91)	0.43	0.39	0.278	1.53 (0.71, 1.31)
TMT B	0.07	0.50	0.890	1.07 (0.40, 2.85)	0.54	0.43	0.209	1.71 (0.74, 3.97)
SCOWA	0.70	0.32	0.030	2.02 (1.07, 3.80)	0.81	0.37	0.028	2.24 (1.09, 4.59)
SVLT-ir	0.38	0.64	0.301	1.45 (0.71, 2.97)	0.60	0.42	0.153	1.81 (0.80, 4.10)
SVLT-dr	0.11	0.377	0.987	0.99 (0.47, 2.07)	0.22	0.54	0.691	1.24 (0.43, 3.60)
SVLT-recog	0.17	0.35	0.622	0.84 (0.42, 1.68)	0.05	0.40	0.891	1.06 (0.48, 2.29)
N (impaired tests)					0.18	0.11	0.094	1.19 (0.96, 1.41)

*Abbreviations: SE – standard error; HR – hazard ratio; TMT A – Visual-motor speed test; TMT B – executive function test; SCOWA – Controlled Oral Word Association Test; SVLT-ir – memory test of immediate recall; SVLT-dr – memory test of delayed recall; SVLT-recog – memory test of recognition.

†Each row presents results of a separate model that included the particular test and two clinical variables as covariates (age and type of surgery).

## Discussion

In our study, verbal fluency score (SCOWA test) was an independent predictor of survival in IDH1-wt and MGMT-unmet patients with high-grade gliomas. Some previous studies conducted both pre- and postoperatively also showed the prognostic value of cognitive functioning. However, not all of these studies identified verbal fluency as the prognostic factor and some yielded significant findings when using other tests (11,12,21). In our study, no other cognitive test was a significant predictor of survival.

Age was significantly associated with the prognosis. This finding is consistent with findings in patients with high-grade glioma. Inconsistent results have been reported regarding the age cut-off for predicting clinical outcomes in glioma patients (22-24). In our study, the limits of the age categories ranged from 50 years, which is also used in recursive partitioning analysis classes (25,26) to 65 years, which is a common cut-off point for age categories.

The extent of resection was found to be an important prognostic factor, with resection and reduction being superior to biopsy (27,28). The extent of resection correlated with survival, even though the exact extent was difficult to determine as not all patients had early postoperative MRI.

While age and the extent of surgery clearly affected survival in this patient group, cognitive functioning had an added value in predicting survival and should not be considered solely as an effect of age or treatment. We suggest that cognitive tests should be included in treatment decision-making, at least as part of frailty screening (29,30).

The limitations of our study are the lack of data on cognitive functioning before surgical treatment and the effect of antiepileptic drugs (as almost all patients received them after surgery). Another limitation is the small sample size, as is the case in many studies involving patients with high-grade gliomas. In addition to the generally low number of newly diagnosed patients with high-grade glioma, the sample size was also limited by a poor performance status and the inability of patients to participate in cognitive function studies (45% in our study), or to complete individual tests. Therefore, we dichotomized the results of the individual cognitive tests using two criteria: *z*≤-1.5 or the inability to complete the test. The latter criterion allowed the patients who were unable to complete a particular test to remain in the analysis. We consider this a strength of our study as it mitigated the overrepresentation of patients with good functioning and prognosis and allowed us to assess the predictive value of cognitive testing with greater validity. The study adds to the knowledge of patients without IDH1 mutation and MGMT methylation, who were less represented in previous studies.

In conclusion, significant prognostic factors for survival in patients with IDH1-wt and MGMT-unmet high-grade gliomas were age and the extent of surgery. After controlling for the influence of these variables, verbal fluency proved to be another significant predictor. Verbal fluency testing is a short and easy-to-administer method that could help clinicians identify candidates for a change in therapeutic approach.
